# Establishment and validation of a multivariate logistic model for risk factors of thyroid nodules using lasso regression screening

**DOI:** 10.3389/fendo.2024.1346284

**Published:** 2024-04-02

**Authors:** Jianning Liu, Zhuoying Feng, Ru Gao, Peng Liu, Fangang Meng, Lijun Fan, Lixiang Liu, Yang Du

**Affiliations:** ^1^ Center for Endemic Disease Control, Chinese Center for Disease Control and Prevention, Harbin Medical University, Harbin, Heilongjiang, China; ^2^ Key Lab of Etiology and Epidemiology, Education Bureau of Heilongjiang Province & Ministry of Health (23618504), Heilongjiang Provincial Key Lab of Trace Elements and Human Health, Harbin Medical University, Harbin, Heilongjiang, China; ^3^ Department of Physical Diagnostics, Beidahuang Industry Group General Hospital, Harbin, Heilongjiang, China

**Keywords:** thyroid nodule, lasso regression, metabolic syndrome, risk factor, logistic regression

## Abstract

**Objective:**

This study aims to analyze the association between the occurrence of thyroid nodules and various factors and to establish a risk factor model for thyroid nodules.

**Methods:**

The study population was divided into two groups: a group with thyroid nodules and a group without thyroid nodules. Regression with the least absolute shrinkage and selection operator (Lasso) was applied to the complete dataset for variable selection. Binary logistic regression was used to analyze the relationship between various influencing factors and the prevalence of thyroid nodules.

**Results:**

Based on the screening results of Lasso regression and the subsequent establishment of the Binary Logistic Regression Model on the training dataset, it was found that advanced age (OR=1.046, 95% CI: 1.033-1.060), females (OR = 1.709, 95% CI: 1.342-2.181), overweight individuals (OR = 1.546, 95% CI: 1.165-2.058), individuals with impaired fasting glucose (OR = 1.590, 95% CI: 1.193-2.122), and those with dyslipidemia (OR = 1.588, 95% CI: 1.197-2.112) were potential risk factors for thyroid nodule disease (p<0.05). The area under the curve (AUC) of the receiver operating characteristic (ROC) curve for the Binary Logistic Regression Model is 0.68 (95% CI: 0.64-0.72).

**Conclusions:**

advanced age, females, overweight individuals, those with impaired fasting glucose, and individuals with dyslipidemia are potential risk factors for thyroid nodule disease.

## Introduction

A thyroid nodule is defined as a mass or lump that forms within the tissues of the thyroid gland (1). Approximately 10-15% of thyroid nodules are malignant, signifying the presence of thyroid cancer (2–7). The incidence of thyroid nodules and thyroid cancer has gradually increased over the past three decades (7–11). This increase may be attributed to advances in diagnostic tools, including high-resolution ultrasound and computerized tomography scans (12–14). Metabolic syndrome is not a distinct disease, instead, it constitutes a clinical syndrome characterized by obesity, diabetes, and dyslipidemia (15). With the improvement of living standards, the nutritional structure of the population has significantly changed. Unhealthy lifestyle habits, including an unhealthy diet and a lack of physical activity, are associated with an increased risk of developing metabolic syndrome (16, 17). The research by Cuneyd Anil et al. found that the risk of developing thyroid nodules significantly increases with an increased body mass index (BMI) and abnormal glucose metabolism (18). Raisa A. Mayers et al. discovered that low levels of high-density lipoprotein cholesterol (HDL-C) and impaired fasting blood glucose are risk factors for nodules (19). A study from China found that elevated levels of triglyceride (TG) are an independent risk factor for thyroid nodules, and the incidence of nodules increases with increasing TG levels (20). Another study from Turkey found that elevated levels of TG and blood glucose are associated with an increased risk of thyroid cancer (21). In addition to the above research, previous studies have also shown that risk factors for thyroid nodules include gender, age, iodine intake, history of radiation exposure, and obesity, among others (22–25). This study analyzed the relationship between thyroid nodules and components of metabolic syndrome and explored the potential associations between thyroid nodules and various metabolic indicators. Our research objective is to develop a risk prediction model for thyroid nodules, investigate the risk factors associated with these nodules, and provide evidence supporting the prevention and early detection of thyroid nodules.

## Materials and methods

### Study subjects

From October 2022 to September 2023, survey questionnaires were distributed to inpatients who underwent thyroid ultrasound and physical examinations at Beidahuang Industry Group General Hospital, located in Harbin, Heilongjiang, China, and written informed consent was obtained from each participant. The questionnaires collected basic demographic information, physical examination data, and biochemical indicators from the hospitalized patients. The inclusion criteria included individuals aged over 18 years, those with relatively complete clinical data, and subjects who underwent biochemical testing in the hospital’s laboratory department. The exclusion criteria were as follows: patients who were pregnant and lactating, those with severe hepatic or renal insufficiency, and those with type 1 diabetes. Research approval was obtained from the Ethics Committee of Harbin Medical University.

### Physical examination and ultrasound examination of the thyroid

Height and weight measurements were taken by professional medical staff with participants being barefoot and wearing light clothing. The formula for calculating BMI is the weight divided by the square of height (kg/m^2^). Before the ultrasound examination of the thyroid gland, the patient is placed in a supine position. The examination is performed by an experienced thyroid ultrasound physician using a transducer with a frequency of 7.5 MHz to determine the size and number of thyroid nodules. A thyroid nodule was defined as a discrete lesion within the thyroid gland that was radio-logically distinct from the surrounding thyroid parenchyma. The criterion for the presence of thyroid nodules is if the nodules have a diameter equal to or exceeding 3 mm (26).

### Laboratory examination

Serum levels of fasting blood glucose (FBG), glycosylated hemoglobin (HbA1c), TG, total cholesterol (TC), HDL-C, low-density lipoprotein cholesterol (LDL-C), apolipoprotein A1 (Apo-A1), apolipoprotein B (Apo-B), lipoprotein(a) (LP(a)), and small dense low-density lipoprotein (sd-LDL) were measured using the fully automated Beckman AU5800 biochemical analyzer.

### Diagnostic assessment criteria for metabolic syndrome, impaired fasting glucose, overweight/obesity, and dyslipidemia

The criteria for defining metabolic syndrome, according to the guidelines of the Chinese Medical Diabetes Association (27), include three or more of the following conditions: 1) Overweight or obesity (BMI ≥ 24 kg/m^2^); 2) Impaired fasting glucose [FBG level ≥ 6.1 mmol/L or postprandial plasma glucose (PPG) level ≥ 7.8 mmol/L]; 3) Systolic blood pressure (SBP) level ≥ 140 mmHg or diastolic blood pressure (DBP) level ≥ 90 mmHg, or treated hypertension; 4) Dyslipidemia is defined as having any one of the following conditions: TC level ≥ 5.2 mmol/L; TG level ≥ 1.7 mmol/L; LDL-C level ≥ 3.4 mmol/L; HDL-C level < 1.0 mmol/L.

### Statistical analysis

The normality of the data was assessed using the Kolmogorov-Smirnov test. Normally distributed continuous variables were presented as mean ± standard deviation (mean ± SD), and compared using an independent samples t-test. Non-normally distributed continuous variables were described using the median of the 25th and 75th percentiles, and compared using the Mann-Whitney U test. Categorical variables were reported as either counts or percentages, and comparisons were made using the chi-square test (χ^2^). The Least Absolute Shrinkage and Selection Operator (Lasso) regression analysis—a shrinkage and variable selection method for linear regression models—was employed. Lasso regression analysis constrains the model parameters, resulting in some variables regression coefficients shrinking to zero. Variables with a regression coefficient that shrank to zero after the shrinkage process were excluded from the model, whereas those with a nonzero coefficient were determined to be strongly associated with the response variable. Incorporating more independent variables beyond a certain threshold did not translate into significant improvements in model performance. Consequently, Lambda.1se was selected to derive a model featuring both excellent performance and a minimal number of independent variables. The Lasso method was employed to analyze the data and select the optimal predictors for the present risk factors. Subsequently, a predictive model was developed employing multivariable logistic regression analysis, which incorporated the selected features from the Lasso regression model. Binary logistic regression analysis was utilized to examine the risk factors and compute the odds ratios (OR) as well as 95% confidence intervals (95% CI) for investigating their relationship with thyroid nodules. The dataset of 1922 research subjects was randomly divided, with 2/3 allocated as the training dataset and 1/3 as the testing dataset. A binary logistic regression model was constructed using the training dataset, followed by validation using the test dataset. Additionally, the receiver operating characteristic (ROC) curve and calibration curve were plotted. Univariate analysis was conducted using SPSS 26. Lasso regression was performed using the “glmnet” package in R 4.2.2. The “rms” package was utilized to build binary logistic regression models and plot calibration curves. The “pROC” package was employed for ROC curve plotting. A p-value <0.05 was considered statistically significant.

## Results

### The univariate analysis of the thyroid nodule group and non-thyroid nodule group

A total of 1922 subjects participated in this study. Among them, there were 1005 people in the thyroid nodule group and 917 people in the non-thyroid nodule group. As shown in **Table 1**, the age of the thyroid nodule group is significantly higher than that of the non-thyroid nodule group. Additionally, the prevalence of thyroid nodules in females is significantly higher than that in the non-thyroid nodule group (p<0.05). BMI, FBG, TG, TC, and HbA1c in the thyroid nodule group were significantly higher than those in the non-thyroid nodule group, while HDL-C in the thyroid nodule group was significantly lower than that in the non-thyroid nodule group (p<0.05). Additionally, there is no statistical difference in the distribution levels of LDL-C, Apo-A1, Apo-B, LP(a), and sd-LDL between the two groups. Overweight, impaired fasting glucose, dyslipidemia, and metabolic syndrome are significantly associated with thyroid nodules (p<0.05), as shown in **Table 1**.

**Table 1:** Comparison between the thyroid nodule group and the non-thyroid nodule group.

Variables	Nodule (-)	Nodule (+)	t (Z;χ^2^)	P
Age (year)	56.05±9.51	59.66±9.24	-8.42	<0.01
Sex			24.56	<0.01
Male	355 (38.71%)	282 (28.05%)		
Female	562 (61.29%)	723 (71.95%)		
BMI (kg/m^2^)	23.68±2.22	24.57±2.03	-9.15	<0.01
FBG (mmol/L)	5.79±0.68	6.12±0.57	-11.39	<0.01
TG (mmol/L)	1.91±0.99	2.29±1.27	-7.43	<0.01
TC (mmol/L)	5.16±0.88	5.52±1.03	-8.36	<0.01
HDL-C (mmol/L)	1.26±0.32	1.05±0.29	14.2	<0.01
LDL-C (mmol/L)	2.78±0.83	2.79±0.86	-0.09	0.925
Apo-A1 (g/L)	1.35±0.21	1.34±0.47	0.52	0.6
Apo-B (g/L)	0.92±0.24	0.93±0.25	-0.72	0.471
LP(a) (mg/L)	142.9(75-236.3)	142.3(75.15-241.3)	-0.19	0.847
sd-LDL (mol/L)	1.00±0.34	1.01±0.44	-0.28	0.781
HbA1c (%)	5.71±0.63	5.84±0.64	-4.56	<0.01
Overweight			28.608	<0.01
No	333 (36.31%)	252 (25.07%)		
Yes	584 (63.69%)	753 (74.93%)		
Impaired fasting glucose			23	<0.01
No	477 (52.01%)	413 (41.09%)		
Yes	440 (47.99%)	592 (58.91%)		
Dyslipidemia			28.61	<0.01
No	413 (45.03%)	333 (33.13%)		
Yes	504 (54.97%)	672 (66.87%)		
Metabolic syndrome			43.46	<0.01
No	763 (83.21%)	708 (70.44%)		
Yes	154 (16.79%)	297 (29.56%)		
Total	917	1005		

BMI, body mass index; FBG, fasting blood glucose; TG, triglyceride; TC, total cholesterol; HDL-C, high-density lipoprotein cholesterol; LDL-C, low-density lipoprotein cholesterol; Apo-A1, apolipoprotein A1; Apo-B, apolipoprotein B; LP(a), lipoprotein(a); sd-LDL, small dense low-density lipoprotein; HbA1c, glycosylated hemoglobin; Nodule (-), non-thyroid nodule group; Nodule (+), thyroid nodule group.

### An analysis of the distribution of thyroid nodule and their risk factors stratified by sex and age

The dataset was stratified by sex and age, and participants were categorized into two age groups: those under 40 years and those 40 years or older. The analysis demonstrated a significantly greater prevalence of dyslipidemia among females aged 40 years or older compared to those under 40 years of age (62% vs. 41%, p < 0.05). Moreover, a significantly higher incidence of thyroid nodules was noted among females aged 40 years or older compared to those under 40 years of age (57% vs. 38%, p < 0.05). Additionally, when evaluated across different sex and age groups, the distribution of other factors associated with thyroid nodular risk did not demonstrate statistical significance, as indicated in **Table 2**.

**Table 2:** Distribution of thyroid nodule and corresponding risk factors categorized by sex and age groups.

Variables	Female			Male		
	<40 year	≥40 year	P	<40 year	≥40 year	P
Overweight			0.588			0.834
No	13 (35%)	381 (30%)		9 (32%)	182 (29%)	
Yes	24 (65%)	867 (70%)		19 (68%)	427 (71%)	
Impaired fasting glucose			0.867			0.251
No	18 (48%)	574 (45%)		10 (35%)	288 (47%)	
Yes	19 (52%)	674 (55%)		18 (65%)	321 (53%)	
Dyslipidemia			<0.05			0.231
No	22 (59%)	484 (38%)		14 (50%)	226 (37%)	
Yes	15 (41%)	764 (62%)		14 (50%)	383 (63%)	
Metabolic syndrome			1			0.648
No	29 (78%)	953 (76%)		23 (82%)	466 (76%)	
Yes	8 (22%)	295 (24%)		5 (18%)	143 (24%)	
Thyroid nodule			<0.05			0.050
No	23 (62%)	539 (43%)		21 (75%)	334 (54%)	
Yes	14 (38%)	709 (57%)		7 (25%)	275 (46%)	

### Variable selection based on lasso regression

**Figure 1:**
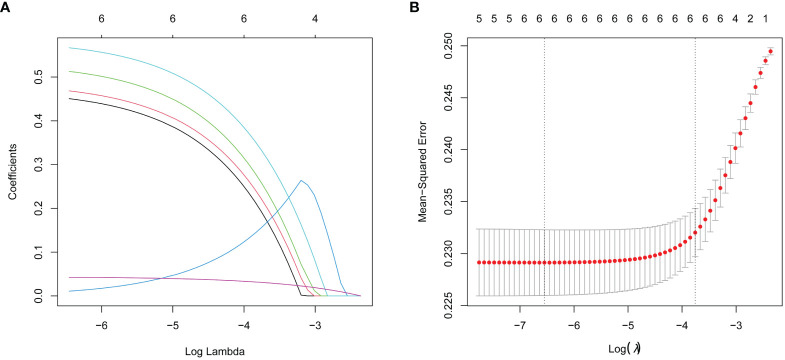
Lasso regression analysis was used to select characteristic factors. **(A)** A coefficient profile plot was produced for the log(lambda) sequence. **(B)** By verifying the optimal parameter (lambda) in the Lasso model, six variables with nonzero coefficients were selected. The Mean-Squared Error curve was plotted versus log(lambda), and dotted vertical lines were drawn based on the 1 standard error criteria using the optimal lambda. Lasso refers to the least absolute shrinkage and selection operator.

The study utilized the presence of thyroid nodules as the dependent variable; categorical variables such as overweight, impaired fasting glucose, dyslipidemia, and metabolic syndrome, along with age and sex, were utilized as independent variables. Lasso regression was subsequently applied to the complete dataset for variable selection. The variable selection process as the λ value changes in the Lasso regression model is shown in **Figure 1**. In **Figure 1A**, the relationship between log(λ) and Lasso regression coefficients is illustrated, showing that as λ increases, the degree of shrinkage in the estimated coefficients of the model’s independent variables increases. The coefficients of the independent variables with a relatively small impact on the dependent variable are shrunk to zero, leading to a reduction in the number of independent variables. **Figure 1B** depicts the curve corresponding to the number of variables as log(λ) changes. The vertical axis represents the model’s mean square error (MSE), the lower horizontal axis represents log(λ), and the upper horizontal axis represents the number of non-zero coefficient independent variables in the model corresponding to different log(λ) values. In **Figure 1B**, the dashed line on the left side represents the optimal tuning parameter λ with the lowest MSE (lambda. min = 0.0014), while the dashed line on the right side represents the λ with MSE within one standard error (lambda.1se = 0.0233). In this study, λ with 1se = 0.0233 was selected as the optimal model. The results show that age, sex, overweight, impaired fasting glucose, dyslipidemia, and metabolic syndrome were retained as variables, as shown in **Table 3**.

**Table 3:** Coefficient table of categorical independent variables selected by Lasso regression.

Variables	Coef (lambda.min=0.0014)	Coef (lambda.1se=0.0233)
Age	0.01	0.01
Sex	0.13	0.08
Overweight	0.12	0.06
Impaired fasting glucose	0.11	0.047
Dyslipidemia	0.11	0.05
Metabolic syndrome	0.001	0.04

### Basic characteristics of variables in the training dataset and testing dataset

The complete dataset was divided into a training dataset consisting of 2/3 of the data and a testing dataset consisting of 1/3 of the data. There is a significant difference in HbA1c between the two groups (p < 0.05), while all other variables show no statistical significance. This indicates that most variables are well-balanced between the two groups, as shown in **Table 4**.

**Table 4:** Basic characteristics of variables in the training dataset and testing dataset.

Variables	Training datasets (1291)	Testing datasets (631)	P	
Age	57.91±9.46	58±9.71	0.844	
Sex			0.462	
Male	435 (33.69%)	202 (32.01%)		
Female	856 (66.31%)	429 (67.99%)		
BMI	24.13±2.17	24.17±2.15	0.716	
FBG	5.97±0.64	5.96±0.65	0.781	
TG	2.13±1.19	2.07±1.10	0.289	
TC	5.36±0.96	5.32±1.01	0.314	
HDL-C	1.15±0.33	1.15±0.31	0.842	
LDL-C	2.80±0.85	2.76±0.84	0.421	
APO-A1	1.34±0.37	1.36±0.37	0.367	
APO-B	0.93±0.25	0.91±0.23	0.211	
LP(a)	144.4(76.3-241.7)	139.6(72.8-230.8)	0.252	
sd-LDL	1.00±0.40	1.02±0.38	0.284	
HbA1c	5.75±0.64	5.82±0.64	0.028	
Overweight			0.828	
No	395 (30.59%)	190 (30.11%)		
Yes	896 (69.41%)	441 (69.89%)		
Impaired fasting glucose			0.937
No	597 (46.24%)	293 (46.43%)		
Yes	694 (53.76%)	338 (53.57%)		
Dyslipidemia			0.315	
No	491 (38.03%)	255 (40.41%)		
Yes	800 (61.97%)	376 (59.59%)		
Metabolic syndrome			0.915	
No	989 (76.61%)	482 (76.38%)		
Yes	302 (23.39%)	149 (23.62%)		
Thyroid Nodule			0.631	
No	611 (47.32%)	306 (48.49%)		
Yes	680 (52.68%)	325 (51.51%)		

BMI, body mass index; FBG, fasting blood glucose; TG, triglyceride; TC, total cholesterol; HDL-C, high-density lipoprotein cholesterol; LDL-C, low-density lipoprotein cholesterol; Apo-A1, apolipoprotein A1; Apo-B, apolipoprotein B; LP(a), lipoprotein(a); sd-LDL, small dense low-density lipoprotein; HbA1c, glycosylated hemoglobin.

### Establishment of the binary logistic regression model

Based on the screening results of Lasso regression, the retained independent variables (age, sex, overweight, impaired fasting glucose, dyslipidemia, and metabolic syndrome) were utilized as predictors, and a binary logistic regression model was constructed using the training dataset, with the presence or absence of thyroid nodules serving as the dependent variable. The results showed that advanced age (OR = 1.046, 95% CI: 1.033-1.060), females (OR = 1.709, 95% CI: 1.342-2.181), overweight (OR = 1.546, 95% CI: 1.165-2.058), impaired fasting glucose (OR = 1.590, 95% CI: 1.193-2.122), and dyslipidemia (OR = 1.588, 95% CI: 1.197-2.112) were risk factors for thyroid nodules (p < 0.05), as shown in **Table 5**.

**Table 5:** Binary logistic regression model of thyroid nodules.

Variables	B	S.E.	Wald	P	OR	OR (95% CI)	
						Lower limit	Upper limit
Age	0.045	0.007	47.610	<0.01	1.046	1.033	1.060
Sex	0.536	0.123	18.748	<0.01	1.709	1.342	2.181
Overweight	0.436	0.145	9.000	<0.01	1.546	1.165	2.058
Impaired fasting glucose	0.464	0.147	9.985	<0.01	1.590	1.193	2.122
Dyslipidemia	0.462	0.145	10.240	<0.01	1.588	1.197	2.112
Metabolic syndrome	-0.027	0.213	0.016	0.90	0.974	0.641	1.478

### Validation of the binary logistic regression model

**Figure 2:**
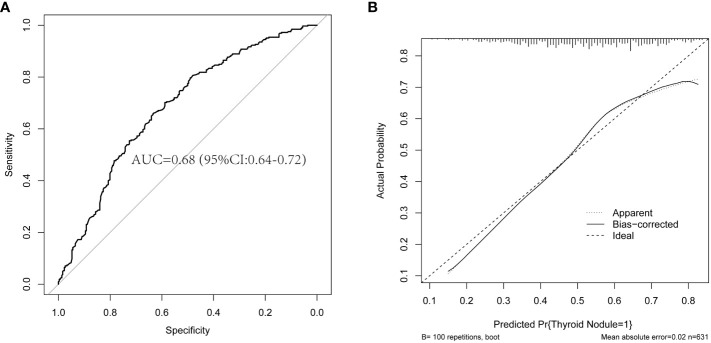
The plots of the ROC curve and calibration curve are based on the binary logistic regression model in the test dataset. **(A)** The ROC curve and AUC are based on the binary logistic regression model in the test dataset. **(B)** The calibration curve is based on the binary logistic regression model in the test dataset. ROC, receiver operating characteristic; AUC, the area under the curve.

To assess the predictive ability of the logistic regression model, we used a test dataset for validation and plotted the ROC curve and calibration curve, as shown in **Figure 2**. The results in **Figure 2A** show that the area under the curve (AUC) is 0.68, with a 95% CI of 0.64-0.72. Furthermore, the results in **Figure 2B** show that both the curve of the current model (Apparent line) and the calibrated curve (Bias-corrected line) exhibit slight fluctuations around the 45-degree diagonal line (Ideal line). The predicted values (with a mean absolute error of 0.02) are overall consistent with the observed variables in the test dataset. The calibration curve indicates that the model has a certain level of calibration ability. This indicates that the binary logistic regression model has a certain ability for discrimination and calibration on the test dataset, and the model demonstrates good predictive performance, as shown in **Figure 2**.

## Discussion

Thyroid nodules have emerged as one of the most common health problems globally (7). With the continuous improvement and modernization of medical equipment, physicians are now able to identify an increasing number of individuals with thyroid nodules (14). Consequently, there has been a significant increase in the prevalence of thyroid nodules over the past three decades (28–30). Thyroid nodules commonly lack noticeable physical symptoms and are frequently detected through routine medical examinations employing thyroid ultrasound (31). Owing to the risk of malignant transformation in 5-10% of thyroid nodules, which indicates a transition from benign to malignant states (6), it is imperative for individuals diagnosed with thyroid nodules to obtain health education, maintain regular check-ups, and monitor thyroid health closely (7, 22, 32). Furthermore, research aimed at identifying potential risk factors contributing to the development of thyroid nodules is essential, as it enables individuals with a healthy thyroid to mitigate risks and safeguard thyroid health—an endeavor of paramount importance. Given that thyroid nodules may represent either a normal inflammatory response or a significant change heralding a tumor, the underlying influencing factors are inherently complex. Prior research has furnished extensive evidence encompassing factors such as sex, age, family history of thyroid disease, radiation exposure during adolescence, and elements like obesity, smoking, alcohol consumption, diabetes, hyperlipidemia, and hypertension (12, 33). In the present study, the subjects consisted of patients from a tertiary-level A hospital in Heilongjiang Province, Northeast China. This region demonstrates a high prevalence of thyroid nodules, and the risk factors that affect the incidence of these nodules are considerably complex. The findings of this study could provide a reasonable basis of reference for further research into the risk factors of thyroid nodules in other regions of China.

The univariate analysis in this study demonstrated that the levels of age, BMI, FBG, TG, TC, and HbA1c were significantly higher in the thyroid nodule group compared to the non-thyroid nodule group; however, the HDL-C level was lower in the thyroid nodule group. In addition, a higher proportion of females, overweight individuals, individuals with impaired fasting glucose, individuals with dyslipidemia, and individuals with metabolic syndrome were significantly more prevalent in the thyroid nodule group compared to the non-thyroid nodule group. Furthermore, the complete dataset was divided into a training dataset, comprising 2/3 of the data, and a testing dataset, comprising 1/3 of the data. Using the variable selection results of lasso regression, a binary logistic regression model was built on the training dataset. The results of the binary logistic regression model showed that advanced age, females, overweight, impaired fasting glucose, and dyslipidemia are risk factors for thyroid nodules. The adjusted model, which accounts for sex and age, demonstrated that females and advancing age represent significant risk factors for the development of thyroid nodules. Additionally, the logistic regression model demonstrates strong predictive abilities, as indicated by the ROC curves and calibration plots in this study. The sex- and age-related differences observed in thyroid nodules in this study align with findings from previous research (3, 20, 22). Women exhibit a higher susceptibility to thyroid nodules, which may be attributed to elevated levels of estrogen (9, 29). Estrogen has been shown to induce the proliferation of thyroid follicular cells, leading to the development of thyroid nodules (2, 17). Although it is known that estrogen plays an important role in the growth of thyroid cells, further investigation is required to understand the underlying mechanisms related to gender differences associated with thyroid nodules (3, 9). With increasing age, thyroid function declines, and fibrosis of the thyroid tissue ensues, which increases the risk of thyroid nodules (8). Another perspective suggests that with aging, the body increases its production of reactive oxygen species and free radicals, ultimately having a detrimental impact on thyroid cells (29).

Obesity and overweight are chronic conditions that have detrimental impacts on overall health (34). In recent years, the prevalence of both obesity and overweight has markedly risen (35, 36). The BMI is considered a crucial indicator for quantifying body fat (37–39). In this study, individuals in the thyroid nodule group had a significantly higher BMI than those in the non-thyroid nodule group. Furthermore, overweight individuals have a 1.5 times higher risk of developing thyroid nodules than individuals with a normal BMI. Research has demonstrated that obese or overweight individuals possess greater quantities of adipose tissue, and the leptin secreted by adipocytes can influence thyroid-stimulating hormone (TSH) levels, thereby impacting thyroid function (28–30). Subsequent research indicates that serum TSH levels are elevated in obese individuals compared to those in the healthy control group, elucidating the association between obesity or overweight and the prevalence of thyroid nodules (27, 40). Impaired fasting glucose is a critical stage in the development of diabetes, and the prevalence of impaired fasting glucose increases with age. Insulin resistance is considered a precursor to impaired fasting glucose (18). Individuals with impaired fasting glucose may exhibit elevated insulin levels, potentially leading to the proliferation of thyroid cells and the formation of thyroid nodules (18, 20, 22). Experimental evidence in rodents indicates that elevated glucose levels are implicated in increasing thyroid oxidative stress. This perspective could provide additional insight into why higher blood glucose levels were observed in the nodal group. In our study, we found that impaired fasting glucose is a risk factor for thyroid nodules. Additionally, the current investigation demonstrates that the levels of FBG and HbA1c in the thyroid nodule group are considerably higher in comparison to those in the non-thyroid nodule group, consistent with previous research (5, 15).

The risk factor for thyroid nodules identified in this study is dyslipidemia, which also constitutes a component of metabolic syndrome and exhibits similarities to obesity and impaired fasting glucose. Extensive research has demonstrated that elevated TG and decreased HDL-C are associated with an increased risk of thyroid nodules (8, 11, 41). In this study, the levels of TG and TC in the group with thyroid nodules were significantly higher than those in the control group, while the levels of HDL-C were significantly lower. These findings align with the findings of previous studies and could further elucidate the role of dyslipidemia as a risk factor for thyroid nodules. Additionally, upon stratification by sex and age, it was found that the prevalence of dyslipidemia was significantly increased in women over the age of 40 years as compared to their counterparts under 40. The incidence of thyroid nodules was likewise significantly higher in women aged 40 and older. This indicates that dyslipidemia is subject to age and sex influences and that a complex association exists between dyslipidemia and thyroid nodules. It is hypothesized that lipid accumulation may induce local inflammation and facilitate the development of thyroid nodules, thereby illustrating a complex interaction between lipid abnormalities and the genesis of thyroid nodules. Furthermore, the underlying mechanisms governing this interaction remain speculative and warrant further investigation. Previous studies have identified metabolic syndrome as a potential risk factor for thyroid nodules (19). In our study, the results of the univariate analysis indicated a significant correlation between metabolic syndrome and thyroid nodules. However, the subsequent multivariate regression analysis revealed no significant association between these two variables. We believe that the primary reason for this phenomenon is the correlation between the individual components of metabolic syndrome, such as overweight, impaired fasting glucose, and dyslipidemia, and the syndrome as a whole (42). This correlation induces collinearity among the variables within the logistic regression model and diminishes the statistical significance of the variable representing metabolic syndrome. Additionally, since metabolic syndrome comprises multiple components, our study only included three of them, potentially resulting in a limited number of patients classified as having metabolic syndrome and presenting challenges for a more precise analysis. Our comprehensive study, which integrates univariate analysis, Lasso regression for variable selection, and logistic regression modeling, provides novel insights into the epidemiology of thyroid nodules. Firstly, the establishment of a predictive model for thyroid nodules holds considerable clinical significance, as it assists clinicians in obtaining a deeper understanding of the risk factors for thyroid nodules, thereby facilitating more informed and accurate decision-making. Additionally, the predictive model is capable of identifying individuals at high risk, thus enabling the implementation of early preventive measures to minimize the incidence of the disease. Furthermore, it can assist hospitals and health departments in the judicious allocation of medical resources amid resource constraints, giving priority to high-risk cohorts. Ultimately, by comprehending and applying the disease predictive model, physicians and public health professionals can more effectively educate the public on associated risks, thereby elevating their self-care awareness and fostering behavioral change.

However, this study has some limitations. Firstly, a logistic regression model was constructed using a training dataset, and the model underwent internal validation using a test dataset. Due to the absence of external validation using data from independent sources, the generalizability of the model remains uncertain, which represents one of the study’s constraints. Secondly, this retrospective observational study is limited to generating etiological hypotheses. Even with rigorous statistical adjustments, the causal relationships remain unproven due to potential influences from undetected biases and confounding variables. If future research includes prospective cohort studies on thyroid nodules, it would yield deeper scientific insights into the risk factors associated with them. Considering the single-center design of this research, it is advisable to consider conducting multi-center studies to enhance the generalizability of the findings. Thirdly, the predictive model of thyroid nodule risk factors developed in this study predominantly comprises variables related to age, sex, and other metabolic indicators. Future research on thyroid nodules ought to contemplate the inclusion of additional potential risk factors, which may encompass dietary habits, genetic predispositions, and histories of environmental exposure. Finally, the participants in this study were relatively older, and the number of included younger participants was limited, which could potentially impact the study findings.

## Conclusions

In conclusion, our study reaffirms the significant roles of age, sex, and various metabolic factors in the risk of developing thyroid nodules. These findings can help stratify higher-risk individuals and inform preventive strategies as well as early interventions. Further research is necessary to elucidate the underlying mechanisms behind these associations and to refine predictive models for the benefit of clinical practice. Consequently, thyroid ultrasound examinations are recommended for females, older individuals, and those with conditions such as overweight/obesity, impaired fasting glucose, and dyslipidemia for the early detection of nodules. Early detection and diagnosis through such screening can facilitate prompt clinical interventions and treatment. In healthy individuals, adopting proactive primary prevention measures, such as maintaining a healthy lifestyle, managing weight, and reducing blood glucose and lipid levels, can help mitigate the risk of developing thyroid nodules.

## Data availability statement

The raw data supporting the conclusions of this article will be made available by the authors, without undue reservation.

## Ethics statement

The studies involving humans were approved by the Ethics Committee of Harbin Medical University. The studies were conducted in accordance with the local legislation and institutional requirements. The participants provided their written informed consent to participate in this study. Written informed consent was obtained from the individual(s) for the publication of any potentially identifiable images or data included in this article.

## Author contributions

JL: Writing – original draft. ZF: Writing – original draft. RG: Writing – review & editing. PL: Writing – review & editing. FM: Writing – review & editing. LF: Writing – review & editing. LL: Writing – review & editing. YD: Writing – review & editing.
